# Assessment of Hepatic Safety of Ulipristal Acetate in Korean Women with Uterine Fibroids: A Real-World Study Using Biochemical Markers

**DOI:** 10.3390/jcm14186496

**Published:** 2025-09-15

**Authors:** Jung Yoon Park, Jeong Namkung, Yong Han Seo, Younjee Chung

**Affiliations:** 1 Department of Obstetrics and Gynecology, Seoul St. Mary’s Hospital, College of Medicine, The Catholic University of Korea, Seoul 06591, Republic of Korea; aurorix86@naver.com; 2 Department of Obstetrics and Gynecology, Eunpyeong St. Mary’s Hospital, College of Medicine, The Catholic University of Korea, Seoul 06591, Republic of Korea; rossa@catholic.ac.kr; 3 Department of Anesthesiology and Pain Medicine, Soonchunhyang University Cheonan Hospital, Cheonan-si 31151, Republic of Korea; c75501@schmc.ac.kr

**Keywords:** ulipristal acetate, uterine fibroids, hepatotoxicity, liver function tests, drug-induced liver injury

## Abstract

**Background/Objectives:** Ulipristal acetate (UPA) is a selective progesterone receptor modulator approved for the treatment of uterine fibroids, but concerns have arisen regarding its potential for severe hepatotoxicity, particularly following regulatory warnings in Europe and Korea. The real-world risk of UPA-induced liver injury in Korean women remains largely unknown. To evaluate the hepatic safety of UPA among Korean women with uterine fibroids using large-scale, population-based healthcare claims and health screening data. **Methods:** A retrospective, propensity score-matched cohort study was conducted using the Korean National Health Insurance Service database. Women diagnosed with uterine fibroids who received UPA between 2013 and 2016 (*n* = 12,166) were compared to matched controls (*n* = 36,498) who did not receive UPA. Primary outcomes included changes in liver enzymes (AST, ALT, γ-GTP) and Fatty Liver Index (FLI) before and after UPA use. **Results:** UPA users showed small but statistically significant increases in ALT, γ-GTP, and FLI compared to pre-treatment values, though all values remained within normal reference ranges. Odds of elevated FLI (≥60) and γ-GTP (≥40 IU/L) were modestly increased in the UPA group, but no cases of severe hepatotoxicity or liver failure occurred. **Conclusions:** UPA use in Korean women was associated with mild, subclinical elevations in liver enzymes and fatty liver index, but not with clinically significant hepatotoxicity. These findings support vigilant hepatic monitoring during UPA therapy, while contextualizing its risk as low in this population.

## 1. Introduction

Uterine fibroids are the most common benign tumors among women of reproductive age, with a global trend of increasing prevalence due to delayed marriage and first pregnancy. Although over 50% of cases remain asymptomatic, symptomatic fibroids can lead to significant morbidity, especially when tumors are large, numerous, or submucosal in location [1]. Among young women with uterine fibroids, preserving fertility is a major concern; treatment options generally include medical therapy or myomectomy.

Conventional medical treatments—such as GnRH agonists, GnRH antagonists, and oral contraceptives—are effective for symptom control, but their impact on fibroid volume is typically temporary and accompanied by adverse effects arising from hypoestrogenism, such as hot flashes and bone loss [2].

Ulipristal acetate (UPA), a selective progesterone receptor modulator (SPRM), was introduced as a novel medical therapy for uterine fibroids, offering sustained fibroid shrinkage, symptom relief, and better tolerability compared to traditional agents [3]. Distinct from existing treatments, UPA demonstrated a more durable reduction in fibroid size that persisted even after the cessation of therapy [4]. Upon its release, UPA received widespread clinical attention due to its non-invasive profile and suitability for long-term use in women wishing to preserve fertility.

However, safety concerns emerged following reports of serious hepatotoxicity in Europe. Among approximately 800,000 UPA users, at least eight cases of severe liver injury requiring liver transplantation were reported. In response, both the European Medicines Agency (EMA) and the Korean Ministry of Food and Drug Safety issued safety warnings and recommendations for liver function monitoring beginning in 2018 [5,6]. Despite these concerns, phase IV post-marketing surveillance data from approximately 3000 Korean women showed no cases of UPA-related hepatotoxicity, and no hepatotoxicity signals were raised during the drug’s development [6].

Nevertheless, additional foreign reports of UPA-induced hepatic injury renewed safety concerns, resulting in further regulatory restrictions and eventual market withdrawal in Korea. To date, however, no cases of severe liver injury or fatal drug-induced hepatotoxicity linked to UPA have been reported in Korean patients. These discrepancies raise the potential influence of interethnic or geographic variation in hepatotoxic risk.

Considering the lack of real-world hepatotoxicity data specific to Korean women, the present study aimed to evaluate the hepatic safety of ulipristal acetate using large-scale, population-based healthcare claims and health screening data.

## 2. Materials and Methods

### 2.1. Data Source and Study Setting

This study utilized data from the Korean National Health Insurance Service (NHIS), which employs ICD-10 codes to document healthcare facility usage, medical interventions, and prescription records for provider reimbursement. The NHIS database comprises demographic details—such as age, sex, and socioeconomic status—comprehensive healthcare service records covering inpatient and outpatient visits, diagnostic codes, treatments, and prescriptions, as well as information on healthcare facilities including their type, location, equipment, and staffing levels, along with mortality data. In addition, the NHIS administers a standardized national health screening program that integrates clinical laboratory assessments with participant-reported surveys on health-related behaviors.

### 2.2. Study Population

**Figure 1:**
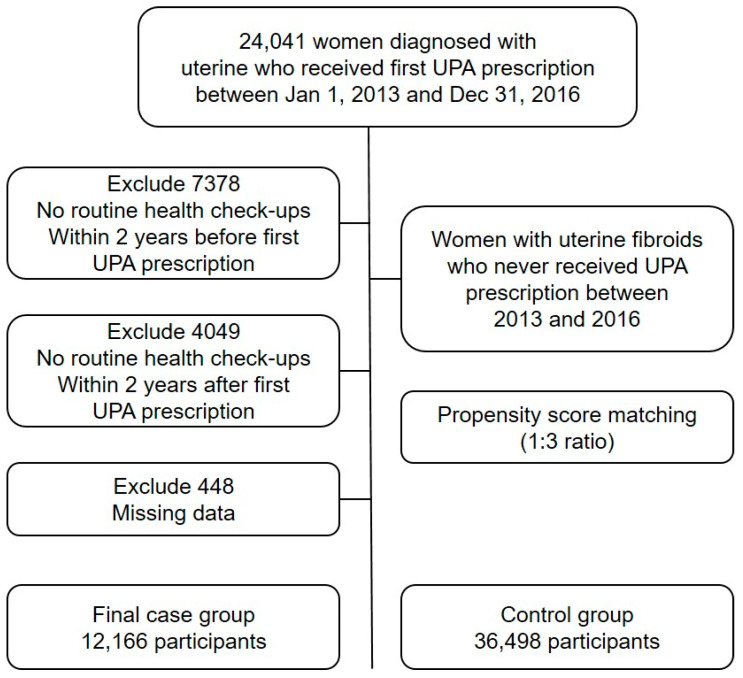
Study flowchart.

We initially identified 24,041 women diagnosed with uterine fibroids who received their first UPA prescription between 1 October 2013 (the first release date of UPA in Korea), and 31 December 2016. From this initial cohort, we applied the following sequential exclusion criteria to ensure data quality and follow-up completeness. First, we excluded 7378 individuals who had not undergone routine health check-ups within two years prior to their first UPA prescription. Second, we removed 4049 individuals who did not complete routine health check-ups within two years after their first UPA prescription. Third, we excluded 448 individuals with missing data essential for our analysis. This systematic exclusion process resulted in a final case group of 12,166 participants who met all inclusion criteria and had complete data for analysis (Figure 1).

For the control group, we identified women who had received medical care for uterine fibroids between 2013 and 2016 but had never been prescribed UPA. To ensure comparability between the case and control groups, we employed 1:3 propensity score matching methodology. This approach allowed us to match each case participant with three control participants based on their propensity scores, which were calculated using baseline characteristics that could influence UPA prescription patterns. The propensity score matching process successfully identified 48,664 control participants, maintaining the predetermined 1:3 ratios and ensuring balanced baseline characteristics between the two groups.

### 2.3. Exposure and Study Outcome

The primary outcome of this study is to analyze changes in liver function tests (AST, ALT, γ-GTP) and Fatty Liver Index (FLI) changes in health screening results between women diagnosed with uterine fibroids who were treated with ulipristal acetate (UPA) and those who were not treated.

When drug-induced liver injury (DILI) is suspected, the hematological tests that primarily serve as indicators include AST, ALT, γ-GTP, and bilirubin. Among these parameters, AST, ALT, and γ-GTP are the tests conducted during national health screenings, and changes in these values were examined.

The Fatty Liver Index (FLI) is a diagnostic model for fatty liver disease that is calculated using four variables: body mass index (BMI), waist circumference, triglycerides, and γ-GTP [7].

The FLI formula is:

$$\mathrm{FLI}\;=\;\left\{1+e^{\left\{0.953\times \backslash ln\left(\left\{triglycerides\right\}\right)+0.139\times BMI+0.718\times \backslash ln\left(\left\{GGT\right\}\right)+0.053\times \backslash \left\{waist\;circumference\right\}-15.745\right\}}\right\}\times 100$$

This index was utilized in the present study because UPA-related case review literature has confirmed that fatty degeneration and steatosis are among the important mechanisms of UPA-induced DILI.

### 2.4. Covariates

The duration of uterine fibroid disease was calculated in years and defined differently for the treatment and control groups. For the ulipristal acetate (UPA) treatment group, the duration was defined as the time from the date of initial diagnosis of uterine fibroids to the date of the first administration of UPA. For the control group, it was defined as the time from the date of initial diagnosis to the date of the first health insurance claim for uterine fibroids within each index year (2013–2016). This approach ensured a consistent measurement framework across both groups.

Smoking status was classified into three categories: non-smoker, ex-smoker, or current smoker. Individuals who consumed 30 g or more of alcohol per day were defined as heavy drinkers. Regular physical activity was characterized as engaging in moderate-intensity exercise for over 30 min at least five times per week, or in vigorous-intensity exercise for more than 20 min at least three times per week.

Body mass index (BMI) was determined by dividing an individual’s weight in kilograms by the square of their height in meters. The presence of comorbid conditions such as diabetes mellitus, hypertension, and dyslipidemia was assessed using ICD-10 diagnostic codes obtained from medical records.

Diabetes mellitus was identified based on either of the following criteria: (1) at least one medical claim with an ICD-10 code of E11–E14 in combination with a prescription for antidiabetic medication within one year, excluding those classified as prediabetic or non-diabetic; or (2) a fasting plasma glucose level of ≥126 mg/dL measured at a health screening, indicating newly diagnosed diabetes.

Hypertension was defined as either (1) a systolic/diastolic blood pressure reading of ≥140/90 mmHg, or (2) at least one claim with a diagnosis coded as I10–I13 or I15, along with a prescription for antihypertensive medication within one year.

Dyslipidemia was identified by either (1) a total cholesterol concentration of ≥240 mg/dL or (2) at least one claim with an ICD-10 code of E78 and a corresponding prescription for lipid-lowering therapy within one year.

Fasting blood samples were obtained for assessment of glucose, lipid profiles, and liver function biomarkers following an overnight fast. All health examinations were conducted at facilities certified by the National Health Insurance Service (NHIS), which are subject to ongoing quality assurance monitoring.

### 2.5. Statistical Analysis

Continuous variables were presented as mean ± standard deviation (SD) and categorical variables as number and percentage. An independent *t*-test was performed for continuous variables, and a chi-square test was conducted for categorical variables.

In this study, we analyzed changes in liver function tests (AST, ALT, γ-GTP) and the Fatty Liver Index (FLI) in health screening data among women diagnosed with uterine fibroids, comparing those treated with ulipristal acetate (UPA) to untreated controls as the primary outcome. To further investigate the clinical significance of these findings, logistic regression analyses were performed for FLI and γ-GTP, as these variables demonstrated significant *p*-values in group comparisons.

Statistical analyses were performed using SAS version 9.4 (SAS Institute Inc., Cary, NC, USA).

A *p* value < 0.05 was considered as statistically significant.

### 2.6. Statement of Ethics

Access to the data was restricted to individuals authorized by the National Health Insurance Service (NHIS, Wonju, Republic of Korea). Permission to use the NHIS data was obtained by submitting a research protocol (REQ000036267-001), which was approved by the Institutional Review Board (IRB) of the Seoul St. Mary’s Hospital (Seoul, Republic of Korea, KC19ZESI0283). Informed consent was not required, as all data had been anonymized and de-identified by the NHIS prior to analysis.

## 3. Results

Baseline characteristics of the study participants are summarized in Table 1. After propensity score matching, a total of 15,864 women diagnosed with uterine fibroids were included, with 12,166 subjects in the UPA (ulipristal acetate) treatment group and 3698 in the control group. Baseline characteristics including age, time since fibroid diagnosis, smoking status, alcohol consumption, regular exercise, and major comorbidities such as diabetes mellitus, hypertension, and dyslipidemia were well balanced between the two groups, with absolute standardized differences (ASD) less than 0.1 for most variables. Key metabolic parameters, including BMI, waist circumference, fasting glucose, lipid profiles, and estimated glomerular filtration rate (GFR), were also comparable between groups.

To evaluate the effect of UPA on liver function and fatty liver index, changes in laboratory parameters before and after UPA administration were assessed and compared to the control group (Table 2). In the UPA group, both the Fatty Liver Index (FLI) and γ-GTP demonstrated significant increases after UPA use compared to baseline, with mean FLI rising from 13.86 ± 16.68 to 16.98 ± 18.86 and γ-GTP from 16.15 (16.16–16.31) to 18 (17.82–18.19), both with *p*-values of 0.0003. ALT also showed a statistically significant increase from 14.95 (14.82–15.08) to 16.54 (16.39–16.69) (*p* = 0.0119). By contrast, AST did not show a significant change after UPA treatment (*p* = 0.0953). Our results showed statistically significant but clinically trivial increases in ALT and γ-GTP, with all values remaining within normal laboratory reference ranges.

To further assess the clinical relevance of these findings, logistic regression analysis was performed for FLI and γ-GTP (Table 3). When applying a threshold of FLI ≥ 60, the odds of elevated FLI were significantly higher in the UPA group compared to controls (OR 1.214, 95% CI 1.084–1.359). Similarly, the odds of having γ-GTP ≥ 40 IU/L were higher in the UPA group (OR 1.154, 95% CI 1.059–1.257). The variable “ γ-GTP high” was defined by dividing γ-GTP levels into quartiles and calculating the odds ratio for belonging to the highest quartile. There was no significant difference in the odds of having γ-GTP in the highest quartile between the UPA and control groups (OR 0.972, 95% CI 0.927–1.019).

## 4. Discussion

In this study, we investigated the effects of ulipristal acetate (UPA) on liver function and metabolic risk in patients with uterine fibroids by evaluating liver enzymes and the fatty liver index (FLI) before and after UPA administration. Our analysis showed that, within the UPA group, post-treatment values of FLI, ALT, and γ-GTP increased significantly compared to pre-treatment values. Our results showed statistically significant but clinically trivial increases in ALT and γ-GTP, with all values remaining within normal laboratory reference ranges.

Notably, the odds of having an FLI of 60 or greater were higher in the UPA group than in the non-UPA group (OR 1.214), and similarly, the odds of having a γ-GTP level equal to or above 40 U/L were elevated in the UPA group (OR 1.154).

The clinical significance of these findings must be considered, especially in the context of drug-induced liver injury (DILI). A γ-GTP level of 40 U/L, while still within normal limits at many laboratories, is at or near the upper reference threshold and may indicate mild hepatic stress or early signs of liver injury, particularly for individuals exposed to potentially hepatotoxic drugs [8,9]. Therefore, even such borderline elevations require careful monitoring. Additionally, FLI ≥ 60 is a widely recognized cutoff indicative of a high probability of hepatic steatosis and, in the context of DILI, may reflect an increased predisposition to drug-induced fatty liver disease, as well as greater metabolic and cardiovascular risk [10,11,12].

In our study, the higher likelihood of exceeding these thresholds in the UPA group suggests that UPA may be associated with subclinical hepatic metabolic stress and an increased risk of developing fatty liver, even if overt abnormalities in liver function tests are not observed.

Importantly, no cases of clinically significant liver injury or liver transplantation were reported during the study period, in contrast to the severe hepatic adverse events observed in some European reports [13,14]. While our findings are tempered by limitations such as the retrospective design, possible discrepancies in the timing of health screening versus UPA administration, and the lack of long-term follow-up data, this study provides meaningful evidence regarding the hepatic safety profile of UPA in the Korean population. It also highlights the importance of vigilant monitoring for even mild elevations in γ-GTP or FLI, as these may signal early hepatic effects and increased metabolic risk in patients receiving UPA.

Recent evidence, including a comprehensive Cochrane review, has further clarified the role of preoperative medical therapy in the management of uterine fibroids [15]. According to this review, preoperative administration of medications—such as gonadotropin-releasing hormone (GnRH) agonists, selective progesterone receptor modulators (SPRMs) like ulipristal acetate (UPA), and other hormone therapies—offers several clinically relevant benefits before surgical intervention. The review concludes that such therapies can effectively reduce fibroid and uterine volume, improve preoperative hemoglobin levels, and decrease intraoperative blood loss, thus potentially making surgery technically easier and reducing transfusion requirements. Additionally, these agents can help correct anemia prior to surgery, which is a common complication in fibroid patients due to chronic heavy menstrual bleeding. However, it is also highlighted that while preoperative therapy can facilitate the surgical process, it does not appear to impact major surgical outcomes such as overall complication rates, need for repeat surgery, or long-term symptom control. The evidence supports the use of preoperative medical therapy primarily as a means to optimize surgical conditions—especially in cases where anemia correction or reduction in fibroid size is desirable prior to myomectomy or hysterectomy [16].

This perspective aligns with our findings in several respects. First, while we identified subclinical changes in liver function and metabolic indices in association with UPA use, the actual rates of clinically meaningful adverse outcomes remained low. The incorporation of preoperative medical therapy, particularly short-term, can be justified by its proven benefit in improving operative conditions without a significant increase in serious risks, particularly when appropriate monitoring is undertaken. Taken together, these results emphasize the importance of individualized patient selection, highlighting the necessity of balancing the surgical benefits of preoperative therapy with the need for vigilant hepatic and metabolic monitoring, especially when agents like UPA are used.

By integrating both our findings and current high-quality evidence from systematic reviews, our study underscores that when used responsibly, preoperative medical therapy is a valuable adjunct that can enhance surgical outcomes without substantially increasing the risk of severe adverse effects for most patients [16].

Selective progesterone receptor modulators (SPRMs) play an important role in the medical treatment of uterine fibroids by targeting progesterone-driven pathways that promote fibroid growth. They selectively modulate progesterone receptors to inhibit fibroid cell proliferation and reduce extracellular matrix accumulation, resulting in significant decreases in fibroid volume and symptoms such as heavy menstrual bleeding [17,18]. Clinically, SPRMs like ulipristal acetate provide rapid symptom relief and are useful both as standalone therapy and as preoperative treatment to improve surgical outcomes. While generally safe, SPRMs require careful monitoring due to potential reversible endometrial changes and rare but serious adverse effects, especially hepatotoxicity associated with ulipristal acetate. Regulatory restrictions in some regions reflect these safety concerns. Nevertheless, when used appropriately with monitoring, SPRMs are effective and valuable options for women seeking to avoid surgery or preserve fertility. Ongoing research aims to develop safer and more effective SPRMs to further improve management of symptomatic uterine fibroids [18].

Recent narrative reviews have reevaluated the hepatic safety profile of ulipristal acetate (UPA) and provide important context for interpreting our findings [19]. Although post-marketing surveillance raised concerns about drug-induced liver injury (DILI) associated with UPA, leading to significant regulatory restrictions, large-scale analyses now suggest that the overall risk of severe DILI with UPA remains low, estimated at 13.5 cases per 100,000 users, with liver transplantation required in only 1 out of 200,000 cases. These incidence rates are in fact lower than those reported for many widely used medications where routine liver function monitoring is not typically mandated. The review also notes that, while strict liver monitoring protocols were imposed for UPA, there remains little evidence that such measures effectively prevent serious DILI events. Consequently, regulatory actions—including indication restrictions, warning labels, mandatory liver function testing, and eventual marketing suspension and withdrawal—may have been disproportionate in comparison to the actual risk. The authors argue for a more balanced approach to DILI management and regulatory oversight, especially when considering that alternative treatments, such as surgery, may present even higher risks. This broader perspective supports the findings of our study, in which we observed only mild, subclinical changes in liver enzyme levels and no cases of severe hepatotoxicity in UPA users. It highlights the ongoing need for proportionate safety measures that preserve patient access to effective therapeutic options, as well as for improved understanding of DILI mechanisms to guide safer prescribing practices and regulatory decisions in the future.

This study represents a large-scale investigation to assess hepatotoxicity associated with ulipristal acetate (UPA) in Korean women through real-world clinical data. Remarkably, we evaluated hepatotoxicity by directly measuring key blood-based liver function parameters, including aspartate aminotransferase (AST), alanine aminotransferase (ALT), gamma-glutamyltransferase (γ-GTP), and the Fatty Liver Index (FLI). These tests are both sensitive and widely recognized markers for detecting liver injury and for elucidating the potential hepatotoxic mechanisms of UPA. In particular, γ-GTP elevation and an increased FLI have been suggested to reflect cholestatic and metabolic injury pathways, which are relevant to the proposed hepatotoxic actions of UPA. Although *p*-values indicated statistical significance, all changes were minor and did not breach normal reference values, suggesting no clinically relevant liver toxicity.

Unlike post-marketing surveillance studies, which typically rely on passive adverse event reporting and may underestimate the true incidence of hepatic effects, our approach incorporated a broader patient population and applied objective, quantitative laboratory data, enabling a more precise and comprehensive evaluation of UPA’s hepatic effects.

Notably, compared to a previous large-scale study in Korean women [20], our study adopted a different methodology to ascertain liver disease. While that study relied solely on International Classification of Diseases (ICD) codes to identify cases of liver disease, we directly assessed liver function using biochemical markers obtained from routine health examinations. This direct measurement allows for the detection of subclinical or early hepatic changes that may be missed when using administrative diagnostic codes alone, resulting in a more clinically meaningful and sensitive evaluation of hepatic safety.

This integration of mechanistic biomarkers with large-scale clinical research represents an important advancement for understanding the safety profile of UPA, strengthening both the validity and the practical relevance of our findings in real-world settings.

This study has several limitations that warrant consideration. First, our analysis did not include data on liver-related symptoms or imaging findings, limiting the ability to detect subclinical or clinically apparent hepatic abnormalities beyond biochemical markers. Second, the relatively short follow-up period precluded evaluation of delayed or long-term hepatotoxicity, an important aspect given that drug-induced liver injury (DILI) may manifest after prolonged exposure or even post-treatment cessation. Third, although we applied propensity score matching to reduce confounding, residual confounding remains possible due to the observational study design. We were unable to adjust for key potential confounders such as preexisting liver disease and concomitant use of hepatotoxic medications, which could influence liver function independently of ulipristal acetate exposure. Finally, while our matching methodology is described with a caliper width of 0.05 for 1:3 matching, sensitivity analyses to test the robustness of our findings were not performed, representing another limitation. Future studies with longer follow-up and more comprehensive clinical data are warranted to further clarify the hepatic safety profile of ulipristal acetate.

In conclusion, this study demonstrates that ulipristal acetate (UPA) use in patients with uterine fibroids is associated with statistically significant but subclinical increases in liver enzyme levels, including γ-GTP around the upper limit of normal, and an elevated Fatty Liver Index (FLI) above 60, indicating mild hepatic metabolic stress and increased risk of fatty liver disease. Despite these biochemical changes, all values remained within normal ranges, and no cases of severe hepatotoxicity were observed, aligning with recent evidence that the risk of serious drug-induced liver injury from UPA is low. Our findings underscore the importance of careful hepatic and metabolic monitoring during UPA therapy while supporting its continued use as a valuable preoperative and medical treatment option for fibroids. These results contribute to a more balanced understanding of UPA’s hepatic safety profile and highlight the need for vigilant yet proportionate clinical management to optimize patient outcomes.

## Figures and Tables

**Table 1:** Baseline Characteristics of the Study Population by Ulipristal Acetate Exposure Before and After Matching.

After Matching	UPA		ASD	Before Matching	UPA		ASD
	No	Yes			No	Yes	
*N*	36,498	12,166		*N*	719,471	12,166	
Age	43.54 ± 7.47	43.54 ± 5.74	0.000123	Age	46.91 ± 8.54	43.54 ± 5.74	0.46365
Time since fibroid diagnosis	2.47 ± 3.55	2.55 ± 3.16	0.023009	Time since fibroid diagnosis	2.02 ± 3.12	2.55 ± 3.16	0.17006
Smoking status			0.006035	Smoking status			0.02103
Non	34,219 (93.76)	11,424 (93.9)		Non	679,142 (94.39)	11,424 (93.9)	
Ex	970 (2.66)	305 (2.51)		Ex	15,549 (2.16)	305 (2.51)	
Current	1309 (3.59)	437 (3.59)		Current	24,780 (3.44)	437 (3.59)	
Alcohol consumption			0.00671	Alcohol consumption			0.11473
Non	23,185 (63.52)	7689 (63.2)		Non	493,772 (68.63)	7689 (63.2)	
Mild	12,804 (35.08)	4298 (35.33)		Mild	216,738 (30.12)	4298 (35.33)	
Heavy	509 (1.39)	179 (1.47)		Heavy	8961 (1.25)	179 (1.47)	
Regular Exercise	6130 (16.8)	2044 (16.8)	0.000147	Regular Exercise	136,462 (18.97)	2044 (16.8)	0.05655
Diabetes	1029 (2.82)	402 (3.3)	0.028152	Diabetes	38,515 (5.35)	402 (3.3)	0.10081
Hypertension	4145 (11.36)	1475 (12.12)	0.023834	Hypertension	129,341 (17.98)	1475 (12.12)	0.16425
Dyslipidemia	3746 (10.26)	1438 (11.82)	0.049671	Dyslipidemia	145,714 (20.25)	1438 (11.82)	0.23135
GFR (mL/min/1.73 m^2^)	97.25 ± 31.43	97.36 ± 25.85	0.003893	GFR (mL/min/1.73 m^2^)	94.27 ± 28.83	97.36 ± 25.85	0.11304
BMI (kg/m^2^)	23.01 ± 3.32	23.04 ± 3.33	0.007961	BMI (kg/m^2^)	23.15 ± 3.23	23.04 ± 3.33	0.03466
Waist Circumference (cm)	74.83 ± 8.3	74.99 ± 8.35	0.019351	Waist Circumference (cm)	75.61 ± 9.71	74.99 ± 8.35	0.06829
Glucose (mg/dL)	92.92 ± 16.6	93.18 ± 16.13	0.015774	Glucose (mg/dL)	94.33 ± 17.84	93.18 ± 16.13	0.06758
Systolic BP (mmHg)	116.42 ± 13.51	116.49 ± 13.43	0.005153	Systolic BP (mmHg)	117.56 ± 14.04	116.49 ± 13.43	0.07723
Diatolic BP (mmHg)	72.8 ± 9.49	72.87 ± 9.57	0.007502	Diatolic BP (mmHg)	73.5 ± 9.65	72.87 ± 9.57	0.0656
Cholesterol (mg/dL)	188.48 ± 31.92	188.66 ± 33.09	0.005389	Cholesterol (mg/dL)	194.7 ± 35.91	188.66 ± 33.09	0.17499
* AST (U/L)	19.81 (19.75–19.87)	19.68 (19.56–19.79)	0.012054	* AST (U/L)	20.97 (20.96–20.99)	19.68 (19.56–19.79)	0.08848
* ALT (U/L)	15.31 (15.24–15.38)	14.95 (14.82–15.08)	0.016936	* ALT (U/L)	16.76 (16.74–16.78)	14.95 (14.82–15.08)	0.10629
* γ-GTP (U/L)	16.26 (16.18–16.35)	16.15 (16–16.31)	0.02282	* γ-GTP (U/L)	17.64 (17.62–17.66)	16.15 (16–16.31)	0.09352
FLI	13.52 ± 15.92	13.86 ± 16.68	0.020536	FLI	15.69 ± 17.33	13.86 ± 16.68	0.10754

Data are expressed as the mean ± SD or n (%); UPA, Ulipristal acetate; ASD, Absolute Standardized Difference; GFR, glomerular filtration rate; BMI, body mass index; BP, blood pressure; AST, Aspartate Aminotransferase; ALT, Alanine Aminotransferase; γ-GTP, Gamma-Glutamyl Transpeptidase; FLI, Fatty Liver Index. * Values are presented as geometric means (95% CI).

**Table 2:** Comparison of Liver Enzyme and Fatty Liver Index Values Pre- and Post-Ulipristal Acetate Administration.

	UPA		*p*-Value
	No	Yes	
FLI			
Pre	13.52 ± 15.92	13.86 ± 16.68	
Post	16.28 ± 18.1	16.98 ± 18.86	0.0003
AST			
Pre	19.81 (19.75–19.87)	19.68 (19.56–19.79)	
Post	20.92 (20.85–20.99)	20.8 (20.68–20.92)	0.0953
ALT			
Pre	15.31 (15.24–15.38)	14.95 (14.82–15.08)	
Post	16.75 (16.67–16.84)	16.54 (16.39–16.69)	0.0119
γ-GTP			
Pre	16.26 (16.18–16.35)	16.15 (16–16.31)	
Post	17.62 (17.53–17.72)	18 (17.82–18.19)	0.0003

UPA, Ulipristal acetate; FLI, Fatty Liver Index; AST, Aspartate Aminotransferase; ALT, Alanine Aminotransferase; γ-GTP, Gamma-Glutamyl Transpeptidase.

**Table 3:** Odds Ratios for Abnormal Hepatic Indices Associated with Ulipristal Acetate Use.

OR (95% CI)	FLI 60	γ-GTP ≥ 40	γ-GTP High
UPA			
No	1 (ref.)	1 (ref.)	1 (ref.)
Yes	1.214 (1.084, 1.359)	1.154 (1.059, 1.257)	0.972 (0.927, 1.019)

FLI, Fatty Liver Index; γ-GTP, Gamma-Glutamyl Transpeptidase; UPA, Ulipristal acetate. γ-GTP high was defined by dividing γ-GTP levels into quartiles and calculating the odds ratio for belonging to the highest quartile.

## Data Availability

Access to the data was restricted to individuals authorized by the National Health Insurance Service (NHIS).
